# 

**Published:** 1877-09-20

**Authors:** 


ORIGINAL AND SELECTED ARTICLES—

Review of Current Neurological Matters. By C. C. Vanderbeck, M.D ........ 227
Case of Epilepsy only during Pregnancy ........ 227
Epileptiform Neuralgia ........ 228
Softening of the Brain ........ 228
Gelseminum Sempervirens in Facial Neuralgia ........ 228
Maternal Impressions. By Geo. A. Dyer, M.D ........ 228
Supra-Pubic Litho.omy. By C. W. Dulles, M.D ........ 230
Hydrastis Canadensis in Uterine Hemorrhage, and Menorrhagia; and also in Dysmenorrhea. By W. A. Gordon, M.D ........ 232
Epithelial Cancer of the Lips and Face ........ 233
Belladonna in Dysentery. By Q. C. Smith, M.D ........ 234
Therapeutic Value of Hydrate of Chloral in Certain Forms of Convulsive Disorder. By Dr. Charles R. Rayne ........ 235

ABSTRACTS AND GLEANINGS—

Dental Irritation, 237; Tuberculosis, Transmissible, 238; Hypodermic Injections of Morphine in the Reduction of Inguinal Hernias, 238; Nocturnal Cramp, 238; Prolapsus Recti, 239; Guarana in Migraine, 239; Tong Pang Chong, 240; The Poison of Small-Pox, 240; Effects of Pilocarpine used Hypodermically, 241; Pityriasis Capitis, 241; Jaborandi in Asthma, 241 Benzoate Lithium in Gout, 241; Gastro-Elytrotomy, 241; To Relieve Morbid Thirst for Alcoholic Drink, 242; Simple Mode of Checking Epistaxis, 242; Protection Against Flies, for Doctors' Horses, 242; A New Preparation of Iodine 242; The Subcutaneous Injection of Morphia with Atropia, 242; Nux Vomica in Opium Poisoning, 243; The Rash Produced by Bromide of Potassium, 243; Retention of Urine, 243; Taynya, a New Remedy for Syphilis and Scrofula, 144; Hydrophobia Cured by a Rattlesnake Bite, 244; Remedy for Hooping-cough, 245; A Means of Arresting Hooping-cough, 245; Pathology of Uræmia and the so-called Uræmic Convulsions, 245; Mercurial Vapor Bath, 246; The Hypophosphites in Phthisis, 246; Chorea, 247; Gonalgia, or Hysterical Arthralgia, 247; What is Chrysarobin, 248; Hydrobromic Acid in Tinnitus Aurium, 248; Acute Ammoniacal Collapse, 249; The Importance of Cicho-Quinine as a Remedy, 249.

PRACTICAL NOTES AND FORMULÆ—

Purpura Hæmorrhagica, 249; Chill Remedy, 249; To Abort a Chill, 249; Prescription for Dysmenorrhea, 249; Arsenic in Chronic Chills, 250; Strychnia, 250; Gonorrhea, 250; For Bronchitis and Asthma, 250; Rheumatism, 250; Irritable Bladder from Acid Urine, 250; Good, Cheap Paste 250; Cold in the Head, 250; Delirium Tremens, 250; Nutritive Enemas 250; In Hemoptysis, 251; Excellent Hydrogogue Cathartic 251; Trismus Nasceitium, 251; Arnica Poisonous, 251; Incontinence of Urine in Children, 251; Vinegar in Opium Poisoning, 252; Opium Poisoning, 252.

SCIENTIFIC ITEMS—

Feeling the Pulse by Telegraph, 252. Oxygen in the Sun, 252. Whitehead Torpedo, 252. Longevity of Man, 252. Petrifaction, 252. Calcium, 252. Jetties, 252. Population of the Globe, 252.

EDITORIAL AND MISCELLANEOUS—

Special Notice, 253. Plain Talk, but no Offense, 253. Medical Schools, 253. Horse Shoeing, 254. A Misfortune, 254. Book Notices, etc.

